# Clinical Characteristics Associated with the PLP-PLS Index, a New Potential Metric to Phenotype Phantom Limb Pain

**DOI:** 10.3390/biomedicines12092035

**Published:** 2024-09-06

**Authors:** Jorge Ortega-Márquez, Justyna Garnier, Lucas Mena, Ana Victoria Palagi Vigano, Eleonora Boschetti Grützmacher, Gabriel Vallejos-Penaloza, Valton Costa, Daniela Martinez-Magallanes, Antonio Vaz de Macedo, Waynice Neiva de Paula-Garcia, Denise Saretta Schwartz, Felipe Fregni, Kevin Pacheco-Barrios

**Affiliations:** 1 Master of Medical Sciences in Clinical Investigation, Harvard Medical School, Boston, MA 02115, USA; 2 Department of Psychology, SWPS University of Social Sciences and Humanities, 03-815 Warsaw, Poland; jgarnier@swps.edu.pl; 3 Hospital das Clínicas, Faculdade de Medicina, Universidade de São Paulo, São Paulo 05508-220, Brazil; lucas.mena@fm.usp.br; 4 Grupo de Ombro e Cotovelo, Faculdade de Medicina do ABC, Santo André 09060-870, Brazil; ana.vigano-2022@ppcr.org; 5 Praxis Am Lichterfelde West, 12205 Berlin, Germany; eleonoraboschetti@hotmail.com; 6 Departamento de Ginecología y Obstetricia, Facultad de Medicina, Clínica Alemana, Universidad del Desarrollo, Santiago 7610315, Chile; gvallejosp@alemana.cl; 7 Departamento de Ginecología y Obstetricia, Hospital Dr Luis Valentín Ferrada, Universidad Finis Terrae, Santiago 7501014, Chile; 8 Neuromodulation Center and Center for Clinical Research Learning, Spaulding Rehabilitation Hospital and Massachusetts General Hospital, Harvard Medical School, Boston, MA 02115, USA; valtoncosta@estudante.ufscar.br (V.C.); dmartinezmagallanes@mgh.harvard.edu (D.M.-M.); fregni.felipe@mgh.harvard.edu (F.F.); 9 Laboratory of Neurosciences and Neurological Rehabilitation, Physical Therapy Department, Federal University of Sao Carlos, Sao Carlos 13565-905, Brazil; 10 Hematology Clinic, Hospital da Polícia Militar, Belo Horizonte, Minas Gerais 30110-013, Brazil; antonio.macedo@ppcr.org; 11 AC Camargo Cancer Center, São Paulo 01509-010, Brazil; waynice.garcia@accamargo.org.br; 12 Departamento de Clínica Médica, Faculdade de Medicina Veterinária e Zootecnia (FMVZ), Universidade de São Paulo, São Paulo 05508-220, Brazil; denise.schwartz@ppcr.org; 13 Unidad de Investigación para la Generación y Síntesis de Evidencia en Salud, Universidad San Ignacio de Loyola, Vicerrectorado de Investigación, Lima 15026, Peru

**Keywords:** phantom limb pain, phantom limb sensation, lower limb amputation, adaptive neuroplasticity

## Abstract

Background: Phantom limb pain (PLP) is highly prevalent after amputation. However, the influence of non-painful sensations (PLS) remains unclear. This study examines the PLP-PLS index as a novel tool to differentiate PLP from PLS and explores the association of clinical factors with the index. Methods: We conducted a cross-sectional analysis of baseline data from 112 participants in a previous factorial trial in patients with unilateral traumatic lower limb amputation. Linear regression models were used to examine the associations between the index and various demographic, psychological and clinical factors. Logistic and Poisson regression, and e-value calculation were utilized for sensitivity analyses. Results: Adjusted multivariable linear regression models demonstrated significant associations of phantom movement sensation (β: −1.532; 95% CI: −2.615 to −0.449; *p* = 0.006) and time since amputation (β: 0.005; 95% CI: 0.0006 to 0.0101; *p* = 0.026) with the PLP-PLS index. These findings were confirmed by multivariable logistic regression (phantom movement sensation OR: 0.469; 95% CI: 0.200 to 1.099, *p* = 0.082; time since amputation OR: 1.003; 95% CI: 1.00003 to 1.007; *p* = 0.048) and sensitivity analyses. Conclusions: Time since amputation and phantom movement sensation likely reflect distinct phenotypes and potential mechanisms for PLP and PLS. The PLP-PLS index is a promising clinical tool for selecting therapies to prevent/treat PLP and for measuring treatment effects to modulate phantom pain. These findings emphasize the importance of understanding the mechanisms underlying PLP and PLS for improving clinical management and guiding future research.

## 1. Introduction

### 1.1. Background

Amputation occurs as a life-saving procedure or when attempts to save the limb are futile, leaving the patient with a physical disability that negatively impacts everyday functioning and quality of life. Lower limb amputation has an estimated worldwide prevalence of 35.3 million people. In the United States, approximately 150,000 patients undergo lower limb amputation each year [1,2]. In Brazil, for example, 43,527 people underwent lower limb amputation from August 2022 to August 2023 [3]. Based on these epidemiological data, understanding the factors linking the demographic, behavioral, biological, and clinical characteristics associated with phantom limb pain is of vital importance in neuroscience and rehabilitation.

A significant factor contributing to the decreased quality of life in amputees is the presence of phantom limb pain (PLP) that is sensed in the missing limb. PLP may occur immediately after amputation and may persist for years [4]. It is also a frequent complaint, with prevalence reported to range from 42.2% to 78.8% in major lower limb amputations [5] and affecting up to 66% of individuals with amputations [4]. Amputees also report experiencing non-painful sensations in the missing limb, referred to as phantom limb sensations (PLS). PLS encompasses a broad spectrum of kinesthetic sensations, such as movements, abnormal size, shape, and position, and exteroceptive sensations that can be perceived as pressure, temperature, itching, electrical sensation, and tingling, among others [6].

While PLP and PLS integrate the clinical spectrum of patients with amputations, and frequently overlap each other, both are also thought to have distinct mechanisms underpinning central and peripheral adaptation processes. PLP is believed to be the result of maladaptive neuroplasticity, that is, an alteration of the normal capacity of the brain to restructure in response to changes, resulting in a dysfunctional adaptation. The absence of sensory inputs from the periphery results in changes in the excitability of sensorimotor areas and in cortical reorganization [7,8]. Thus, PLP may result from the maladaptive reorganization of the sensorimotor cortical representation of the amputated limb, leading to increased excitability of the corticospinal pathways due to the reduced motor threshold. The involvement of the motor areas associated with descending pain modulation may also account for the processes underpinning the chronification of pain [9]. On the other hand, PLS seems to be more related to somatosensory overexcitation resulting from deafferentation; hence, it is thought to reflect a neuroplasticity compensatory process of brain adaptation to compensate for the absence of peripheral sensory inputs [10]. Importantly, the presence and intensity of PLS, especially the sensation of movement, have been associated with better profiles of adaptation and less PLP intensity after amputation [11].

A recent approach to differentiate PLP and PLS from a clinical perspective is the use of the PLP-PLS index, which is calculated by subtracting PLS intensity from PLP intensity [6]. This index was reported as a potential tool to specifically evaluate phantom limb pain without the influence of other phantom phenomena, but also to characterize clinical sub-phenotypes within patients (i.e., based on different ratios of PLP and PLS). Additionally, based on the distinct mechanisms of these sensations, the PLP-PLS index can be used to estimate the level of the sensorimotor maladaptive process (positive values indicating maladaptation, wherein pain prevails over sensation) and the level of compensatory sensorimotor processes (with zero or negative values, in which phantom sensations are equal to or higher than pain).

### 1.2. Significance

The PLP-PLS index stands as a promising clinical tool for assessing adaptation after amputation and measuring the effectiveness of treatments aimed at reducing phantom limb pain. The characteristics of the index may represent a key aspect in integrating not only non-painful sensations, but also other aspects reported to influence phantom limb pain, such as behavioral, psychological, and clinical factors [4,10,12,13,14,15,16,17]. However, analyses exploring the association of these factors with the index are currently lacking.

### 1.3. Aim of the Study

In this study, we attempt to explore the PLP-PLS index as a potential phenotyping tool to advance the understanding of the role of non-painful sensations and other clinical aspects in phantom limb pain. Therefore, we aim to explore the demographic, psychological, and clinical factors associated with the index and its modification in patients with phantom limb pain after traumatic lower limb amputation.

## 2. Methods

### 2.1. Study Design

This study is a cross-sectional exploratory analysis of baseline data collected from a randomized factorial trial assessing the effects of transcranial direct current stimulation (tDCS) and mirror therapy (MT) in patients with unilateral traumatic lower limb amputation [6]. A sample size of 132 (adjusted for a 20% attrition rate) was calculated. A total of 132 patients were enrolled between 5 July 2015 and 30 March 2020, in two sites located at Spaulding Rehabilitation Hospital, Boston, MA, USA, and the University of São Paulo, São Paulo Brazil. Twenty patients were not randomized as they withdrew from the trial, which resulted in 112 subjects being randomly assigned to 1 of 4 study groups [6,18].

### 2.2. Study Population

This trial included individuals aged 18 years or older, with chronic PLP lasting for at least 3 months (experienced at least once a week) after complete healing of the residual limb, reporting at least 4 out of 10 on a Visual Analog Scale (VAS), using no pain medications or on a stable dosage for at least 2 weeks prior to enrollment. The study population did not include oncological, congenital, and non-traumatic patients due to underlying differences in the neuroplasticity profiles. Additional exclusion criteria were: self-reported alcohol or illicit drug abuse over the past 6 months, contraindications to tDCS (ferromagnetic metal or implanted electronic medical devices in the head or neck); unstable medical conditions, history of head injury with permanent neurological deficits, uncontrolled epilepsy or prior seizures within the last year, unexplained fainting spells or loss of consciousness during the last 2 years, pregnancy, history of neurosurgery or mirror therapy within 3 months prior to enrollment.

### 2.3. Outcomes

#### 2.3.1. Demographics and Clinical Characteristics

Demographic variables (age, gender, study site) and clinical characteristics were collected from the original study baseline data. Clinical information included the side of amputation, level of amputation, time since amputation, and history of opioid, gabapentin, pregabalin, common analgesics, antidepressants, and anticonvulsants intake.

#### 2.3.2. Painful and Non-Painful Sensations

Pain was assessed by the standardized VAS scale, in which participants rated pain level from 0 (no pain at all) to 10 (worst pain ever felt). For an easier identification of the correct response, both PLP and PLS scales were colored to introduce a visual indicator, transitioning from green at 0 to red at 10. Data also included the presence of pain before amputation. The following non-painful sensations (PLS) were measured individually: phantom limb movement sensation (kinetic sensation of the amputated body part) telescoping (distal part of the phantom gradually felt to approach the residual limb), itching, abnormal shape, abnormal position, something touching, warmth, cold, and electric sensations. These questionnaires measuring pain and non-painful sensations were chosen based on previous research trials assessing subjects with phantom limb pain [8,19,20,21].

#### 2.3.3. Psychological Assessments

The Beck Depression Inventory (BDI) and the Beck Anxiety Inventory (BAI) were used for the psychometric assessment in the original study design of the trial, based on their previous use in subjects with phantom limb pain and other chronic pain conditions [22,23,24]. The BDI assesses the presence of several symptoms related to depression, such as irritability, hopelessness, and decreased cognitive performance [25]. On the other hand, the BAI was used to measure the symptoms of anxiety felt by an individual over the past week (e.g., numbness, tingling, sweating, and fear of the worst happening) [26].

#### 2.3.4. Primary Outcome

**Figure 1:**
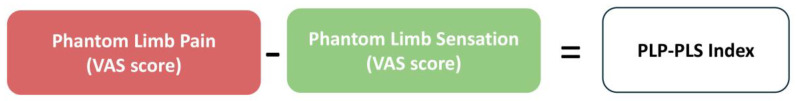
Calculation of PLP-PLS index.

**Figure 2:**
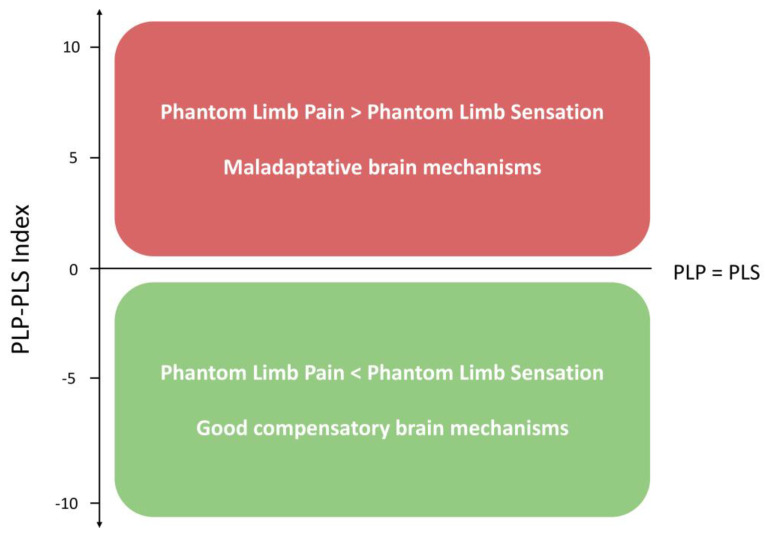
Interpretation of PLP-PLS index based on brain organization mechanisms after amputation.

The PLP-PLS index was the main outcome of this study, as we intended to test the original variable. The index was calculated as a difference between PLP and PLS. Both PLP and PLS were measured on a VAS at baseline. PLP was rated from 0—indicating no pain at all, to 10—indicating the worst pain imaginable. PLS was assessed from 0—no phantom limb sensation, to 10—most intense sensation ever felt in the phantom limb. Graphical representations of the calculation and interpretation of the PLP-PLS index are depicted in Figure 1 and Figure 2 for illustrative purposes.

#### 2.3.5. Secondary Outcome

In order to test the robustness of the index, we calculated the PLP/PLS ratio. For this purpose, we categorized this variable into a binary outcome where values lower than 1 were coded as 0 and those higher as 1 (bi-index).

### 2.4. Statistical Analysis

To conduct this cross-sectional analysis, we included all the 112 participants reported in the baseline data in the original study. Statistical analysis was conducted using Stata statistical software (version 18; StataCorp., College Station, TX, USA). To evaluate normality of the continuous variables, visual evaluation of histograms, skewness, kurtosis, and the Shapiro–Wilk test were used. For baseline descriptive statistics, we used mean and standard deviation (SD) to summarize continuous variables and frequency and percentages for categorical data.

#### 2.4.1. Primary Analysis

We used linear regression models as a simple but efficient method to test and quantify the association of linear dependent variables such as the PLP-PLS index [27]. First, we performed univariate analyses to assess the individual associations of independent variables with our dependent variable, the PLP-PLS index. This process allowed an initial understanding of potential associations and helped identify potential confounders. Subsequently, multivariable models were used to test the association of different independent variables at the same time, which allows us to estimate the effect of each independent variable while holding the values of all other independent variables constant [28]. This was based on the following assumptions: linearity in the relationship with the independent factors, normality of the index distribution, and homoscedasticity. We hypothesized that baseline characteristics are associated with the PLP-PLS index and that they have different association factors. Before constructing regression models, directed acyclic graphs (DAGs) were built to visually represent the assumptions about the statistical relationships between baseline characteristics and the PLP-PLS index (Supplementary Figure S1). Next, in order to test the association of biological, physiological and clinical factors with the index, we conducted univariate linear regression analyses followed by an iterative forward selection process to generate a multivariable linear regression model. Beta coefficients were considered for the effect size measure in the models. The significance level, denoted as alpha, was established at 0.05. To complete the final model, we tested the exclusion or inclusion of individual variables, considering the current literature on biological adaptive mechanisms related to phantom limb painful and non-painful sensations. To test the assumptions of the model, Q-Q plots and residual analysis were performed. In addition, collinearity statistics and Akaike’s information was calculated to estimate prediction errors and, thereby, the quality of the model.

#### 2.4.2. Sensitivity Analysis

In order to test the robustness of our linear regression model, the following sensitivity analyses were performed:

(A) Logistic regression model.

With the aim of enhancing the exploration of the categorical nature of the PLP-PLS index in classifying patients based on the presence or absence of phantom limb pain, we employed a logistic regression methodology. We conducted univariate and multivariable logistic regression analyses to calculate odds ratios (ORs) (effect size measure) for subjects exhibiting a positive or negative value of the PLP-PLS index. In this case, the index was used as a categorical (dichotomous) variable, with the median as a cutoff (0 = negative PLP-PLS index < 0; 1 = positive PLP-PLS index ≥ 0). We incorporated adjustments for variables consistent with those in our main linear regression model. Additionally, we computed the Area Under the Curve (AUC) as an aggregated metric to assess the overall performance of the final logistic regression model.

(B) Poisson regression model.

To elaborate further on our data analysis robustness methodology, we utilized a Poisson regression to calculate adjusted prevalence ratios (PRs) (effect size measure) with robust variance adjustment. This choice was based on the potential overestimation of effect size typically associated with odds ratios in logistic regression, due to their non-collapsible nature [29]. The independent variable in this analysis was the PLP/PLS ratio, representing the PLP divided by PLS and dichotomized to categorized neuroplasticity as either maladaptive or compensatory following amputation.

(C) E-value calculation.

To specifically assess robustness to confounding in our analyses, an E-value was calculated for the most significant factor observed in the multivariable regression models. We used ORs from the final logistic model as effect size measures for this calculation. The E-value indicates the minimum strength of association that unmeasured confounders would need to have with both independent and dependent variables to potently nullify the observed association [30].

## 3. Results

### 3.1. Sample Characteristics

We analyzed 112 patients with lower limb amputation. Among them, 65.2% (*n* = 73) were from Brazil and 34.8% (*n* = 39) from the United States; 66.1% were male and 33.9% female. The mean age of the patients was 44 (±14.8) years. Other demographic characteristics are described in Table 1.

### 3.2. Linear Regression Analysis

After descriptive analyses and normality tests, the PLP-PLS index showed a normal distribution (Supplementary Figure S2). In the univariate linear regression analysis (Supplementary Table S1), time since amputation had a positive association (β: 0.005; CI: 0.0006 to 0.010; *p* = 0.02), while phantom limb movement sensation (β: −1.338; CI: −2.384 to −0.292; *p* = 0.01) and anxiety (β: −0.054; CI: −0.0548 to −0.0002; *p* = 0.04) were negatively associated with the PLP-PLS index. When building the preliminary multivariable regression model, the aforementioned variables (with the exception of anxiety) remained with significant *p*-values (Supplementary Table S2). Based on previous studies and literature evidence [6,11,16], adjustments were made for gender and gabapentin for the final multivariable regression model (Table 2). For this model, time since amputation was associated with a higher index (β: 0.005; CI: 0.0006 to 0.010; *p* = 0.02), while phantom limb movement sensation (β: −1.532; CI: −2.615 to −0.449; *p* = 0.006) and anxiety (β: −0.055; CI: −0.109 to −0.0004; *p* = 0.048) were associated with a lower index. Gender and gabapentin remained with non-significant values (*p* > 0.5). Linear regression assumptions were met based on Q-Q plots and residual analysis (Supplementary Figure S3). In addition, collinearity statistics were calculated, and no correlation between independent variables was found (Supplementary Table S3). Akaike Information Criteria (AIC) was calculated and compared between the preliminary (AIC = 519) and final (AIC = 521) multivariable linear regression models for the PLP-PLS index (treated as a continuous variable) (Supplementary Table S4).

### 3.3. Sensitivity Analysis

(A) Logistic Regression Analysis.

In the univariate logistic regression analysis (Supplementary Table S5), adjusting for *p* ≤ 0.1, we found the following significant variables: side of amputation (OR: 2.19; CI: 1.010–4.748, *p* = 0.046), phantom limb sensation of cold (OR: 2.11; CI: 0.868–5.154, *p* = 0.098), time since amputation (OR: 1.003; CI: 0.999–1.007, *p* = 0.071), anxiety (OR: 0.961; CI: 0.924–0.999, *p* = 0.049), and phantom limb movement sensation (OR: 0.529; CI: 0.244–1.147, *p* = 0.1). To build the final logistic regression model (Table 3), the considerations were time since amputation, phantom limb movement sensation, and anxiety, adjusted for the same additional variables (gender and gabapentin). For this model, time since amputation (OR: 1.003; 95% CI: 1.00003 to 1.007; *p* = 0.048) showed higher odds of a positive PLP-PLS index with each month since amputation. The presence of phantom limb movement sensation exhibited a notable trend—though not statistically significant—towards higher odds of a negative PLP-PLS index (OR: 0.469; CI: 0.200 to 1.099; *p* = 0.082). Gender, anxiety and gabapentin remained with non-significant values (*p* > 0.1). The AUC of the model reported a value of 0.681 (Supplementary Figure S4).

(B) Poisson Regression Analysis.

The results of the Poisson regression model (Supplementary Table S7) indicate that individuals with phantom limb movement sensation are 0.562 times more likely to have a PLP/PLS ratio of 1 or greater than those without phantom limb movement sensation, yielding a PR of 0.562 (95% CI: 0.373 to 0.848, *p*-value: 0.006; E-value for point estimate: 2.96 and for confidence interval: 1.64) when adjusting for the same factors presented in the linear regression model.

(C) E-value calculation.

We estimated the E-value (for point estimate: 31.6, CI: 4.17) for the effect of the phantom limb movement sensation variable (coefficient −1.53, SE 0.55) in the final multivariable linear regression model (Table 2). The E-value showed that with a coefficient of −1.53 (95% CI: 2.03–8.86) for phantom limb movement sensation, an unmeasured confounder that was associated with both the outcome and the exposure by a coefficient of 31.6-fold each, above and beyond the measured confounders, could explain away the estimate, whereas weaker joint confounder associations could not. A graphical representation of the E-value is presented in Supplementary Figure S5.

## 4. Discussion

### 4.1. Main Findings

In this study, we aimed to explore the relationship between clinical, psychological, and demographic factors and the PLP-PLS index. Overall, our results based on adjusted regression models and sensitivity statistical analysis have indicated the PLP-PLS index is associated mainly with time since amputation and phantom limb movement sensation. The longer the time since amputation the higher the PLP-PLS index. Additionally, the presence of phantom limb movement sensation was associated with a lower PLP-PLS index. As mentioned before, higher values of this index indicate the prevalence of phantom pain over non-painful phantom sensations; on the contrary, lower values of the index indicate that the person experiences more phantom sensations than pain. Cumulative evidence suggests that these two phenotypes may be related to distinct neurophysiological mechanisms, with important clinical implications [7,8,31,32,33].

### 4.2. Significance and Implications

**Figure 3:**
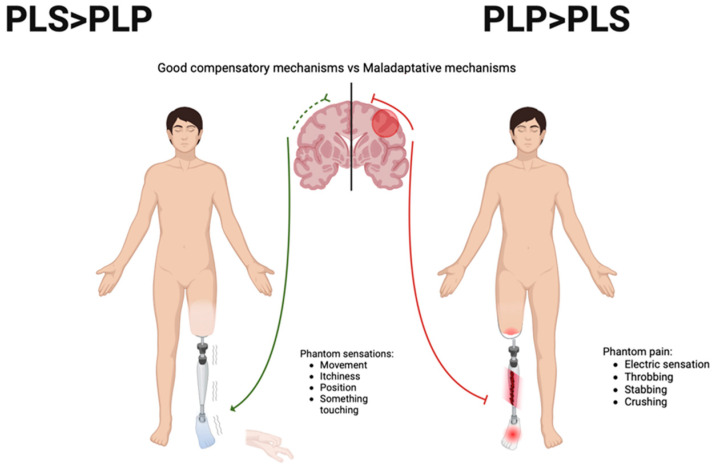
Clinical interpretation of PLP-PLS index based on brain organization mechanisms after amputation.

Time since amputation may reflect the maladaptive neuroplastic process leading to the chronification of PLP, especially in the absence of corrective or adaptive processes that may be time dependent [11,34]. Therefore, it suggests that a higher PLP-PLS index could be used to estimate not only the level of pain dissociated from non-painful PLS but also to reflect the level of neuroplastic maladaptation. Conversely, when phantom movement sensation is present without PLP, as observed before, it may reflect a better compensatory neuroplastic process in sensorimotor circuits involved with modulation of pain [10] (Figure 3). Studies have demonstrated that patients who reported being able to feel and move their phantom limbs were also found to experience less phantom pain and that loss of motor control over the phantom limb is associated with increased pain [11,19,35]. This discrepancy between phantom movement (but not other phantom sensations) and phantom pain likely reflects the dissociation of pathophysiological mechanisms generating these phenomena [19]. Moreover, it has been suggested that this protective characteristic of phantom limb movement sensation is the potential explanation for the effectiveness of movement-inducing therapies, such as mirror therapy, sensorimotor training, and biofeedback, for decreasing PLP.

It is known from previous animal and human studies that, after amputation, robust cortical reorganization takes place, inducing changes in cortical excitability. These neurophysiological mechanisms for pain involve alterations in the adaptive and compensatory processes in the primary and secondary sensorimotor cortical areas and broad areas of the brain, such as the lateral prefrontal cortex, thalamus, and anterior cingulate cortex [36,37,38]. Changes in motor cortex excitability seem to play a major role in the development of PLP by reduced excitability after amputation, as well as alterations in the activity of brain areas involved in the descending pain inhibitory system, demonstrated by the indirect modulation of this area by long-term excitatory M1 tDCS, which was shown to reduce PLP [8,19,39]. Consequently, reinforcement of the cortical representation of the amputated limb might be key to compensate for the maladaptive plasticity that induces pain [19,40].

In other amputation etiologies, pain before amputation, level of amputation, number of surgeries, lower limb amputation, and proximal amputation level were described as risk factors for PLP [41,42,43]. Moreover, research has also shown that, for patients with traumatic lower limb amputation, pain before surgery and level of amputation seem to be predictors of residual limb pain (RLP) but not PLP, which highlights the potential divergent neurophysiological mechanism of phantom pain [43]. Interestingly, in the present study, these variables were not associated with the PLP-PLS index.

Cultural factors may play a significant role in the perception and management of PLP as previously reported in other studies [11,44]. For example, in particular, the middle-aged patients observed in this study or different behavioral aspects such as attitudes towards health, pain tolerance, and seeking medical help can influence how individuals experience and report pain [45]. Moreover, cultural factors significantly influence the perception, expression, and management of pain and psychological symptoms [4,12,22]. These cultural factors influence and shape individuals’ experiences and responses to pain, affecting both diagnosis and treatment outcomes [46]. Therefore, recognizing these cultural dimensions provides a more comprehensive understanding of the patient experience and informs more culturally sensitive approaches to care.

### 4.3. Strengths and Limitations

Our study has some strengths and limitations. Among its strengths, the novel aspect of exploring the PLP-PLS index and its relationship to clinical manifestations and to demographic and psychological factors gave us a better understanding of the neuroplasticity and reorganization roles in the pathophysiology of PLP. Furthermore, this novel tool could contribute to explaining possible neuroplasticity-based mechanisms underpinning movement-inducing therapies that can potentially benefit patients with traumatic lower limb amputation by considering both PLS and PLP.

As for the limitations of our study, the rather specific nature of the study sample, i.e., the fact that only patients with lower limb amputations, a traumatic etiology, and moderately intense pain at a chronic stage were included, could induce selection bias and reduce the external validity of the results, thus undermining the generalizability of our findings, for instance, to patients with non-traumatic causes of amputation (e.g., cancer, diabetes, infection), in which pain might have been experienced before the amputation and the adaptive processes might differ. Furthermore, given the cross-sectional design of our study, longitudinal research would need to be conducted to better explore the behavior of the PLP-PLS index over time and the factors associated with phantom limb manifestations. Lastly, an additional downside, given the novelty of the PLP-PLS index, is the uncertainty around the most suitable analysis method to use. While, in our study, the use of a frequentist statistical framework with multivariable regression analyses allowed us to test simple but understandable relationships with the index, other approaches can be used. For instance, Bayesian methods could also improve the understanding of such relationships by integrating prior clinical knowledge and evidence, potentially revealing more about the probabilities of different outcomes associated with the index [47,48,49]. Thus, further research using the PLP-PLS index is necessary to better explore the impact and interpretation of this index as a tool for measuring different aspects associated with PLP, phenotyping patients based not only on painful sensations, and assisting in the clinical decision-making process of treatment selection and evaluation.

## 5. Conclusions

The PLP-PLS index is a sound tool to differentiate phantom pain from non-painful phantom limb sensations. Moreover, it can be used to differentiate two phenotypes of patients: those with a predominance of phantom pain and maladaptive cortical neuroplastic processes from those with less pain and a predominance of phantom limb sensations, likely reflecting a better neuroplastic compensatory process to the loss of the limb. Hence, such clinical characteristics associated with the PLP-PLS index seem to reflect two distinct phenotypes of patients with amputations: those with prevalent pain/less phantom movement vs. those with prevalent sensations/less pain.

In this study, we explored the associations between clinical, psychological, and demographic variables and the PLP-PLS index, successfully achieving our main objective. The PLP-PLS index emerges as a promising tool to measure pure PLP among other phantom phenomena, differentiate subsets of patients after amputation (especially in light of the effect of time since amputation on phantom pain phenotypes), select the most likely effective therapies to prevent or treat PLP, and measure the effect of treatments aimed to increase pain modulation and compensatory central processes.

Previous studies have highlighted the difference between phantom pain and phantom sensations, suggesting disparate causes, mechanisms, and clinical implications. We emphasized the association between the PLP-PLS index and variables likely reflecting these divergent mechanisms related to both these phantom phenomena (painful and non-painful sensations). In fact, the associations found in this study highlight two potential mechanisms underlying PLP and PLS: the effect of maladaptation on the chronification of pain and maintenance of the pain process, as opposed to the apparently protective compensatory process related to phantom movement sensation.

Furthermore, this study underscores the potential clinical value of the index. First, it may enable the differentiation of patient phenotypes post-amputation, incorporating both painful and non-painful sensations, as well as diverse cultural and psychological aspects, thus offering a more comprehensive clinical evaluation of these individuals. Second, our findings on phantom movement sensation associated with the absence or non-predominance of PLP may suggest a therapeutic target of movement-inducing therapies to foster compensatory sensorimotor processes, potentially modulating PLP. Therefore, we recommend further investigation and exploration of this index as a clinical tool to distinguish subsets of patients and enhance the understanding of the neuroplastic mechanisms underlying phantom limb phenomena. This could ultimately aid in identifying individual needs, treatment effects, as well as new therapeutic targets based on more comprehensive clinical evaluation of patients with phantom limb pain after amputation.

## Figures and Tables

**Table 1:** Descriptive statistics of the study sample.

Variables	N = 112
Site (Brazil)	73 (65.2%)
Age	44.3 (14.8)
Gender (male)	74 (66.1%)
Side of amputation (right)	65 (58.0%)
Level of amputation (below knee)	57 (50.9%)
Depression (BDI scale)	9.31 (8.37)
Anxiety (BAI scale)	10.929 (10.4)
Time since amputation (months)	74.89 (114.24)
Pain prior to the amputation (Yes)	73 (65.2%)
Phantom limb pain (PLP) intensity on VAS (0–10)	6.08 (1.7)
Telescoping sensation (Yes)	6.04 (2.9)
Itching sensation (Yes)	66 (61.1%)
Abnormal shape sensation (Yes)	23 (21.3%)
Abnormal position sensation (Yes)	17 (15.7%)
Something touching sensation (Yes)	25 (23.1%)
Warmth sensation (Yes)	27 (25.0%)
Cold sensation (Yes)	27 (25.0%)
Electric sensation (Yes)	70 (64.8%)
Abnormal position sensation (Yes)	17 (15.7%)
Phantom limb movement sensation (Yes)	55 (50.9%)
Opioid intake (Yes)	18 (16.1%)
Gabapentin intake (Yes)	24 (21.4%)
Pregabalin intake (Yes)	5 (4.5%)
Common analgesics intake (Yes)	13 (11.6%)
Antidepressants intake (Yes)	22 (19.6%)
Anticonvulsants intake (Yes)	2 (1.8%)

BDI, Beck Depression Inventory; BAI, Beck Anxiety Inventory; PLP, phantom limb pain; VAS, Visual Analog Scale.

**Table 2:** Final multivariable linear regression model for the PLP-PLS index as a continuous variable adjusted for gender, time since amputation, phantom limb movement sensation, anxiety, and gabapentin intake.

PLP-PLS Index (Continuous) 107 Observations, Adjusted R^2^ = 9.41%			
Variable	Coefficient	95% CI	*p*-Value
Gender (female)	0.750	−0.485, 1.987	0.231
Time since amputation	0.005	0.0006, 0.0101	0.026
Phantom limb movement sensation	−1.532	−2.615, −0.449	0.006
Anxiety (BAI)	−0.055	−0.109, −0.0004	0.048
Gabapentin intake	0.444	−0.813, 1.703	0.485

**Table 3:** Final multivariable logistic regression model for the PLP-PLS index as a categorical variable adjusted for gender, time since amputation, phantom limb movement sensation, anxiety, gabapentin intake.

PLP-PLS Index (Categorical) 107 Observations, Pseudo R^2^ = 7.2%			
Variable	Odds Ratio	95% CI	*p*-Value
Gender (female)	0.946	0.361, 2.479	0.910
Time since amputation	1.003	1.00003, 1.007	0.048
Phantom limb movement sensation	0.469	0.200, 1.099	0.082
Anxiety (BAI)	0.966	0.924, 1.011	0.147
Gabapentin intake	0.701	0.260, 1.889	0.483

## Data Availability

The original contributions presented in the study are included in the article/Supplementary Materials, further inquiries can be directed to the corresponding author.

## Supplementary Materials

The following supporting information can be downloaded at: https://www.mdpi.com/article/10.3390/biomedicines12092035/s1, Table S1: Univariate linear regression analysis; Table S2: Multivariable linear regression analysis (preliminary model); Table S3: Collinearity statistics for multivariable linear regression analysis (final model); Table S4: Comparison of Akaike Information Criteria (AIC) for the preliminary and final multivariate linear regression models; Table S5: Univariate logistic analysis; Table S6: Multivariable logistic regression analysis (preliminary model); Table S7: Poisson regression analysis. Figure S1: Directed acyclic graph clinical characteristics; Figure S2: Histogram PLP-PLS index; Figure S3: Quantile-Quantile (Q-Q) plot for the final multivariable linear regression model; Figure S4: Area under Curve (AUC) for final multivariable logistic regression model; Figure S5: E-value calculation phantom limb movement sensation; Figure S6: Scatter plot of PLP-PLS index by gender adjusted using the final multivariable linear regression model; Figure S7: Scatter plot of PLP-PLS index over time since amputation, adjusted using the final multivariable linear regression model; Figure S8: Scatter plot of PLP-PLS index by phantom limb sensation, adjusted using the final multivariable linear regression model; Figure S9: Scatter plot of PLP-PLS index by anxiety (BAI Scale), adjusted using the final multivariable linear regression model; Figure S10: Scatter plot of PLP-PLS index by gabapentin, adjusted using the final multivariable linear regression model.
