# Supplementary material for: Clinical Characteristics Associated with the PLP-PLS Index, a New Potential Metric to Phenotype Phantom Limb Pain
Source: Biomedicines. 2024 Sep 6;12(9):2035. doi: 10.3390/biomedicines12092035 (PMC11429012; doi:10.3390/biomedicines12092035)
Supplement: Supplementary file 1 [file biomedicines-12-02035-s001.zip › biomedicines-3110639-supplementary.pdf]

Clinical Characteristics Associated with the PLP-PLS Index, a New Potential Metric to Phenotype Phantom Limb Pain

SUPPLEMENTARY MATERIAL

List of Tables

- Supplementary Table S1. Univariate linear regression analysis.
- Supplementary Table S2. Multivariable linear regression analysis (preliminary model).
- Supplementary Table S3. Collinearity statistics for multivariable linear regression analysis (final model).
- Supplementary Table S4. Comparison of Akaike Information Criteria (AIC) for the preliminary and final multivariate linear regression models.
- Supplementary Table S5. Univariate logistic analysis.
- Supplementary Table S6. Multivariable logistic regression analysis (preliminary model).
- Supplementary Table S7. Poisson regression analysis.

List of Figures

- Supplementary Figure S1. Directed acyclic graph (DAG) clinical characteristics – PLP-PLS index.
- Supplementary Figure S2. Histogram PLP-PLS index (treated as a continuous variable).
- Supplementary Figure S3. Quantile–Quantile (Q-Q) plot for the final multivariable linear regression model.
- Supplementary Figure S4. Area Under Curve (AUC) for final multivariable logistic regression model.
- Supplementary Figure S5. E-value calculation phantom limb movement sensation.
- Supplementary Figure S6. Scatter plot of PLP-PLS index by gender, adjusted using the final multivariable linear regression model.
- Supplementary Figure S7. Scatter plot of PLP-PLS index over time since amputation, adjusted using the final multivariable linear regression model.
- Supplementary Figure S8. Scatter plot of PLP-PLS index by phantom limb sensation, adjusted using the final multivariable linear regression model.

- Supplementary Figure S9. Scatter plot of PLP-PLS index by anxiety (BAI scale), adjusted using the final multivariable linear regression model.
- Supplementary Figure S10. Scatter plot of PLP-PLS index by gabapentin, adjusted using the final multivariable linear regression model.

TABLES

Supplementary Table S1. Univariate linear regression analysis of clinical and demographic variables associated with PLP-PLS index as a continuous variable.

Variable	β-coefficient	95% CI	p-value
Site (Brazil)	-0.749	-2.064, 0.564	0.21
Age	0.033	-0.009, 0.077	0.08
Gender (female)	-0.038	-1.310, 1.232	0.95
Side of amputation (right)	0.878	-0.296, 2.053	0.13
Level of amputation (above knee)	-0.306	-1.456, 0.843	0.59
Depression (BDI scale)	-0.025	-0.0941, 0.043	0.46
Anxiety (BAI scale)	-0.054	-0.109, -0.0002	0.04
Time since amputation (months)	0.005	0.0006, 0.010	0.02
Pain before amputation (yes)	-1.210	-2.396, -0.023	0.04
Itching sensation (yes)	-0.460	-1.551, 0.630	0.40
Abnormal shape sensation (yes)	0.413	-0.887, 1.714	0.53
Abnormal position sensation (yes)	-0.681	-2.140, 0.778	0.35
Something touching sensation (yes)	-0.178	-1.443, 1.086	0.78
Warmth sensation (yes)	-0.116	-1.348, 1.116	0.85
Cold sensation (yes)	0.525	-0.702, 1.754	0.39
Electric sensation(yes)	-0.152	-1.269, 0.964	0.78
Phantom limb movement sensation (yes)	-1.338	-2.384, -0.292	0.01
Opioid intake (yes)	0.590	-0.972, 2.154	0.45
Gabapentin intake (yes)	0.553	-0.845, 1.952	0.43
Pregabalin intake (yes)	-2.685	-5.014, 0.055	0.05
Common analgesics Intake (yes)	1.275	-0.505, 3.055	0.15
Antidepressants intake (yes)	0.371	-1.075, 1.818	0.61
Anticonvulsants intake (yes)	-0.015	-4.361, 4.330	0.99

Supplementary Table S2. Preliminary multivariable linear regression model for PLP-PLS index (treated as a continuous variable) on time since amputation, phantom limb movement sensation, anxiety.

PLP-PLS index (continuous)<br>107 observations, adjusted R^2= 9.29%			
**Variable**	**Coefficient**	**95% CI**	**p-value**
Time since amputation	0.005	0.0002, 0.009	0.038
Phantom limb movement sensation	-1.441	-2.499, -0.382	0.008
Anxiety	-0.039	-0.089, 0.009	0.116

Supplementary Table S3. Collinearity statistics for final multivariable linear regression model for PLP-PLS index (treated as a continuous variable) adjusting for gender, time since amputation, movement sensation, anxiety, gabapentin intake.

Collinearity Statistics		
**Variable**	**VIF**	**1/VIF**
Gender (female)	1.31	0.763
Time since amputation	1.06	0.942
Phantom limb movement sensation	1.12	0.889
Anxiety	1.25	0.800
Gabapentin intake	1.03	0.975

Supplementary Table S4. Comparison of Akaike Information Criteria (AIC) for the preliminary and final multivariate linear regression models for PLP-PLS index (treated as a continuous variable).

Model Fit Measures								
						**Overall Model Test**		
**Model**	**R**	**R^2**	**Adjusted R^2**	**AIC**	**F**	**df1**	**df2**	**p-value**

Preliminary	0.344	0.119	0.0929	519	4.62	3	103	0.005
Final	0.370	0.137	0.0941	521	3.20	5	101	0.010

Supplementary Table S5. Univariate logistic analyses of clinical and demographic variables associated with PLP-PLS index treated as a categorical (dichotomous) variable.

Variable	Odds Ratio	95% CI	p-value
Site (Brazil)	1.074	0.492, 2.342	0.856
Age	1.009	0.984, 1.035	0.465
Gender (female)	0.617	0.279, 1.367	0.235
Side of amputation (right)	2.190	1.014, 4.732	0.046
Level of amputation (above knee)	0.863	0.410, 1.813	0.698
Depression (BDI scale)	0.981	0.937, 1.027	0.422
Anxiety (BAI scale)	0.961	0.924, 0.999	0.049
Time since amputation (months)	1.003	0.999, 1.006	0.071
Pain before amputation event (yes)	0.570	0.260, 1.254	0.161
Itching sensation (yes)	0.736	0.337, 1.608	0.443
Abnormal shape sensation (yes)	1.415	0.559, 3.579	0.463
Abnormal position sensation (yes)	0.816	0.284, 2.345	0.707
Something touching sensation (yes)	0.752	0.301, 1.874	0.541
Warmth sensation (yes)	0.777	0.320, 1.889	0.579
Cold sensation (yes)	2.115	0.871, 5.133	0.098
Electric sensation (yes)	1.448	0.647, 3.240	0.365
Phantom limb movement sensation (yes)	0.529	0.244, 1.147	0.10
Opioid intake (yes)	1.136	0.412, 3.130	0.805
Gabapentin intake (yes)	0.747	0.298, 1.869	0.534
Pregabalin intake (yes)	0.264	0.028, 2.444	0.241
Common analgesics intake (yes)	1.92	0.583, 6.281	0.281
Antidepressants intake (yes)	1.142	0.447, 2.916	0.780
Anticonvulsants intake (yes)	1.115	0.067, 18.517	0.939

Supplementary Table S6. Preliminary multivariable logistic regression model for PLP-PLS index as categorical variable adjusted for time since amputation, phantom limb movement sensation, and anxiety.

PLP-PLS index (categorical)<br>107 observations, Pseudo R^2 = 6.8%			
**Variable**	**Odds Ratio**	**95% CI**	**p-value**

Time since amputation	1.003	1.0001, 1.007	0.044
Phantom limb movement sensation	0.476	0.207, 1.095	0.081
Anxiety	0.965	0.926, 1.005	0.094

Supplementary Table S7. Poisson regression model for PLP/PLS ratio (PLP divided by PLS). The model is adjusted for gender, time since amputation, phantom limb movement sensation, anxiety, gabapentin.

**PLP-PLS index (categorical)<br>107 observations, Pseudo R^2= 3.2%**			
**Variable**	**PRR**	**95% CI**	**p-value**
Gender (female)	1.0152	0.651, 1.583	0.07
Phantom limb movement sensation	0.562	0.372, 0.847	0.006
Time since amputation	1.001	1.001, 1.002	0.033
Anxiety	0.991	0.923, 1.014	0.416
Gabapentin intake	1.034	0.684, 1.561	0.873

FIGURES

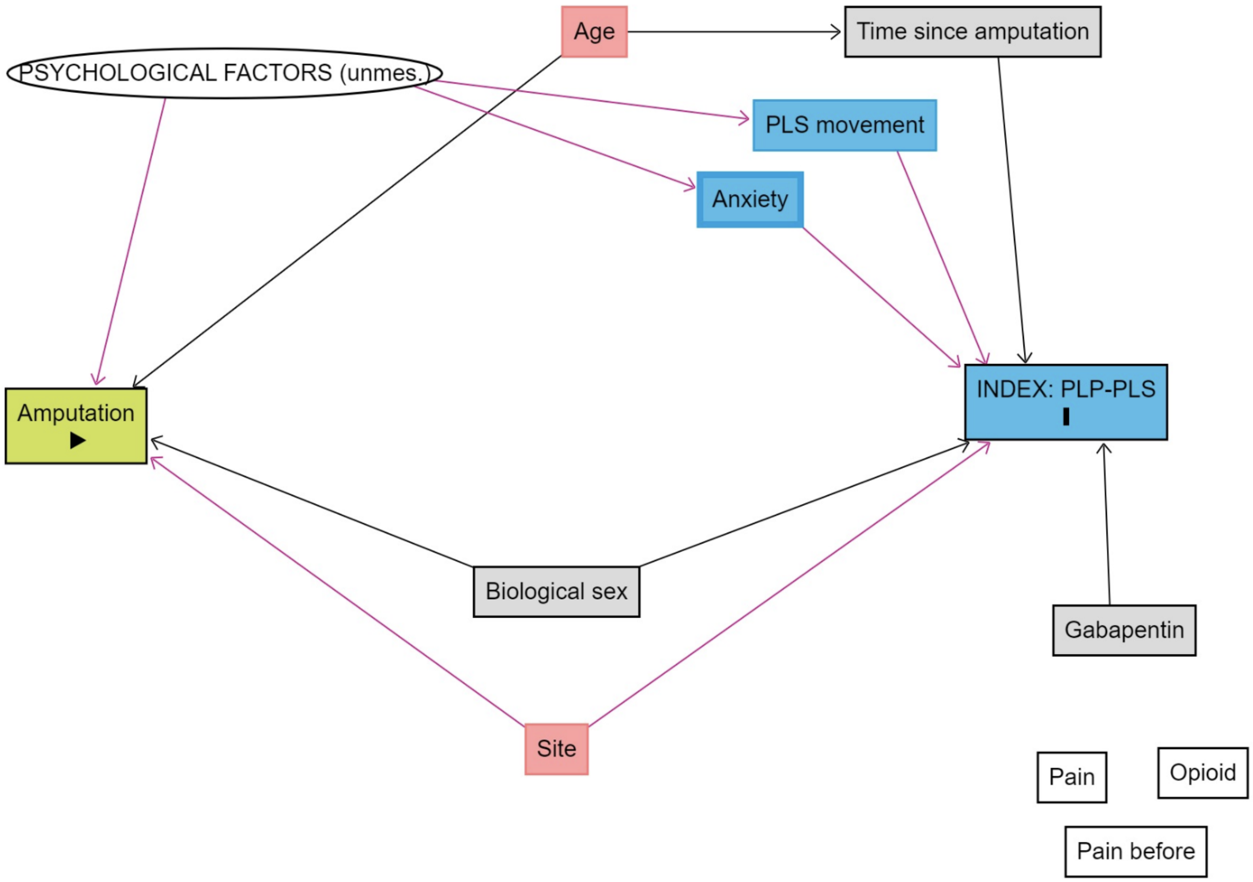

Supplementary Figure S1. Directed acyclic graph (DAG) visualizing potential statistical relationships between baseline clinical characteristics and PLP-PLS index.

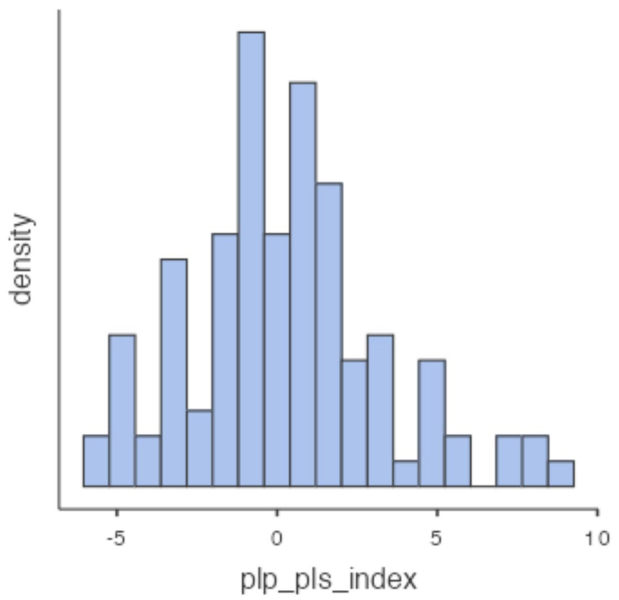

Supplementary Figure S2. Histogram of PLP-PLS index distribution as a continuous variable.

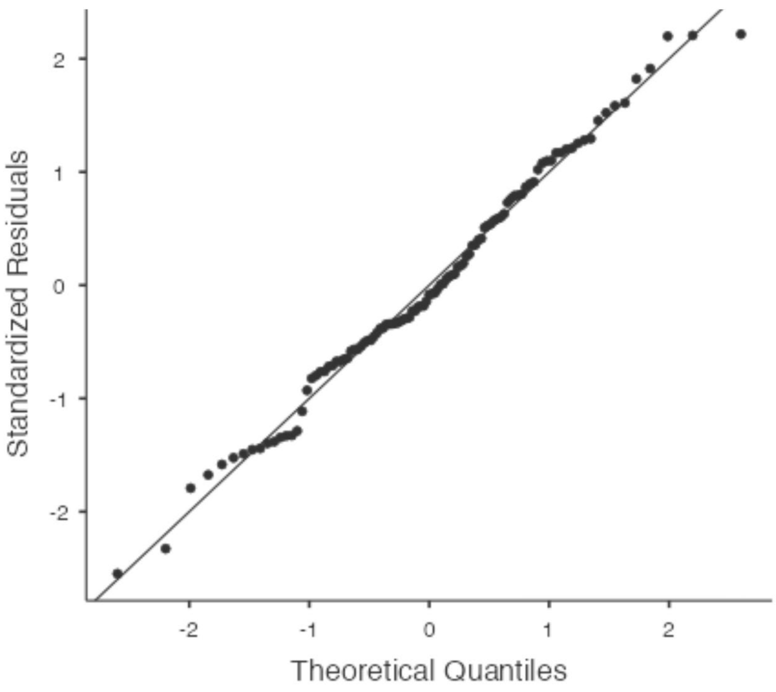

Supplementary Figure S3. Quantile–Quantile (Q-Q) plot evaluating the normality of residuals for the final multivariable linear regression model of the PLP-PLS index (treated as a continuous variable). This model adjusts for gender, time since amputation, movement sensation, anxiety, gabapentin intake.

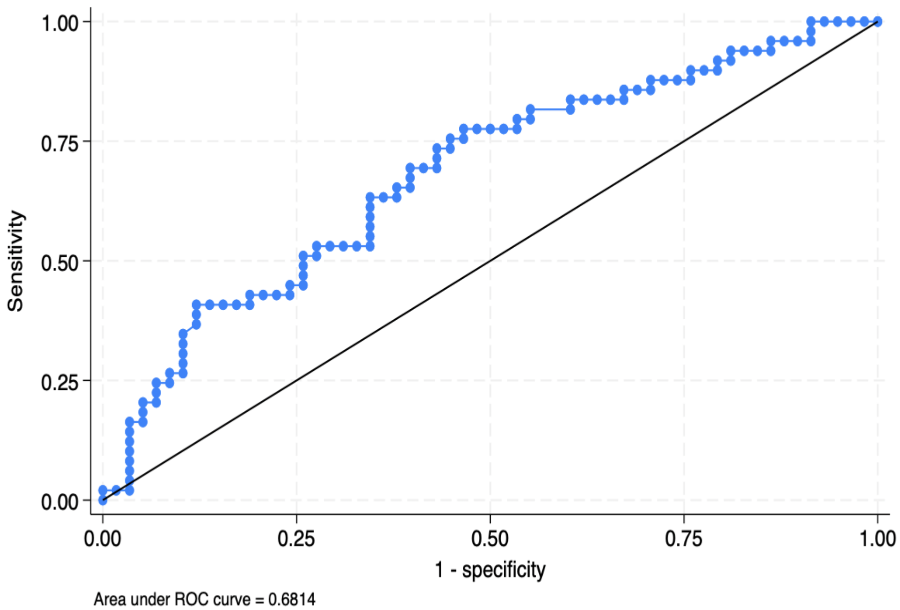

Supplementary Figure S4. Area Under Curve (AUC=0.681) for final multivariable logistic regression model adjusting for gender, time since amputation, phantom limb movement sensation, anxiety, and gabapentin intake.

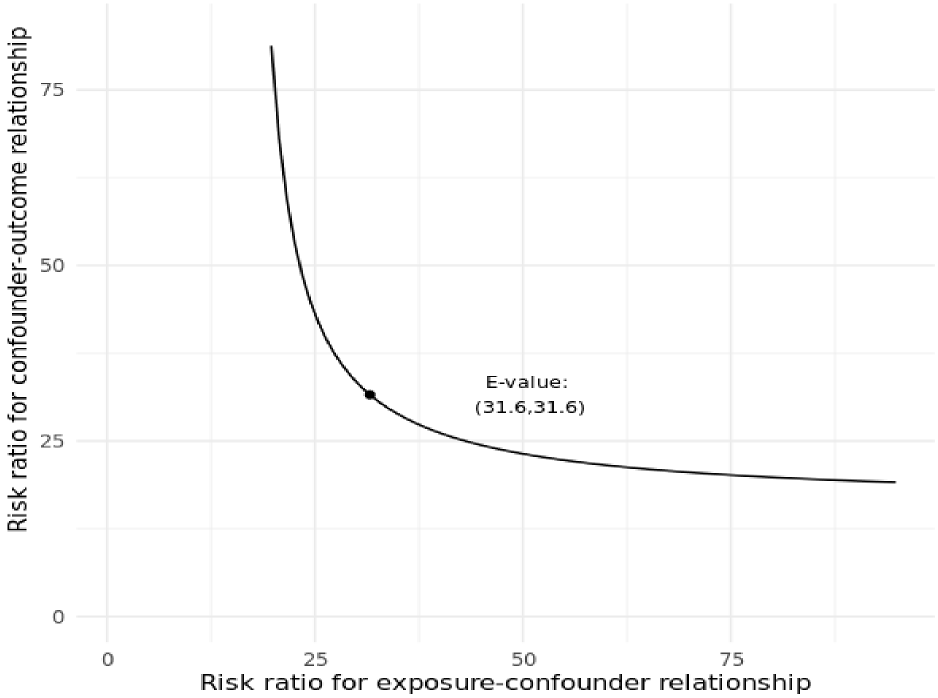

Supplementary Figure S5. E-value calculation phantom limb movement sensation.

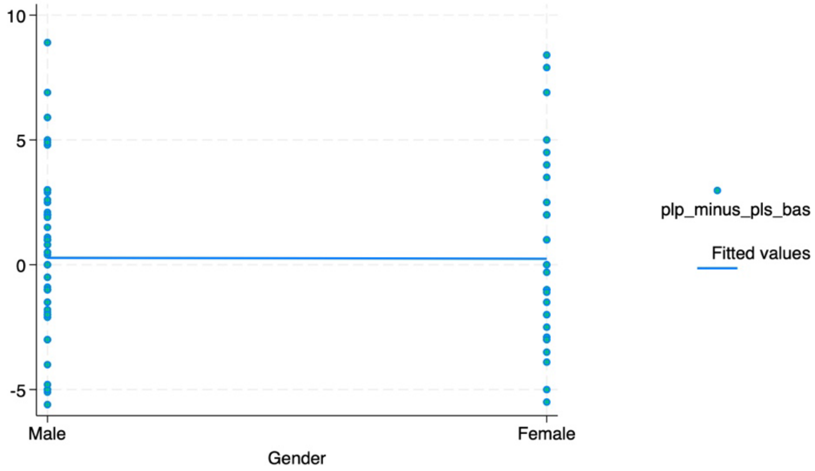

Supplementary Figure S6. Scatter plot of PLP-PLS index by gender, adjusted using the final multivariable linear regression model.

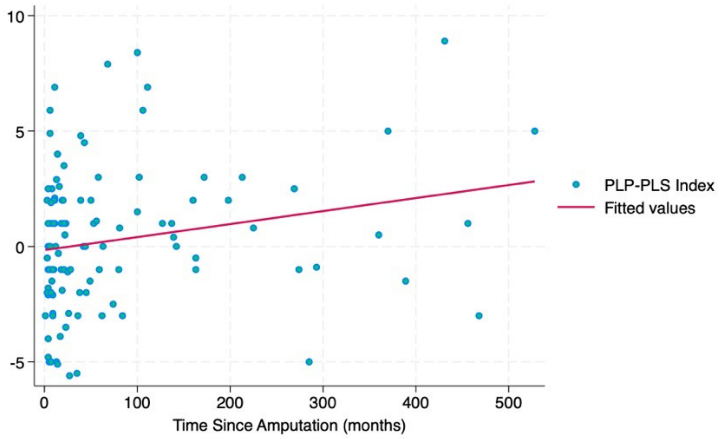

Supplementary Figure S7. Scatter plot of PLP-PLS index over time since amputation, adjusted using the final multivariable linear regression model.

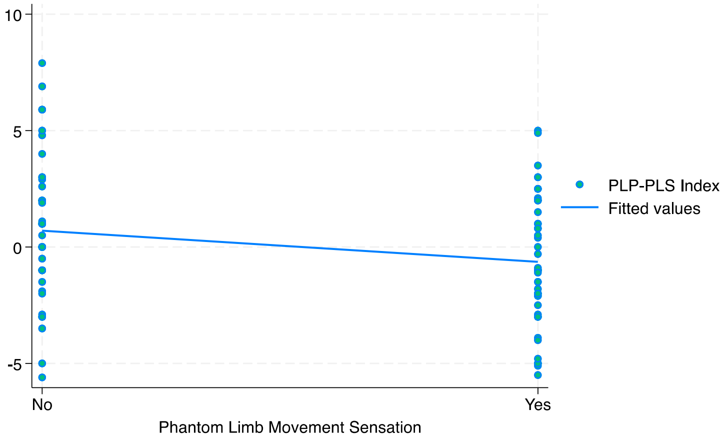

Supplementary Figure S8. Scatter plot of PLP-PLS index by phantom limb sensation, adjusted using the final multivariable linear regression model.

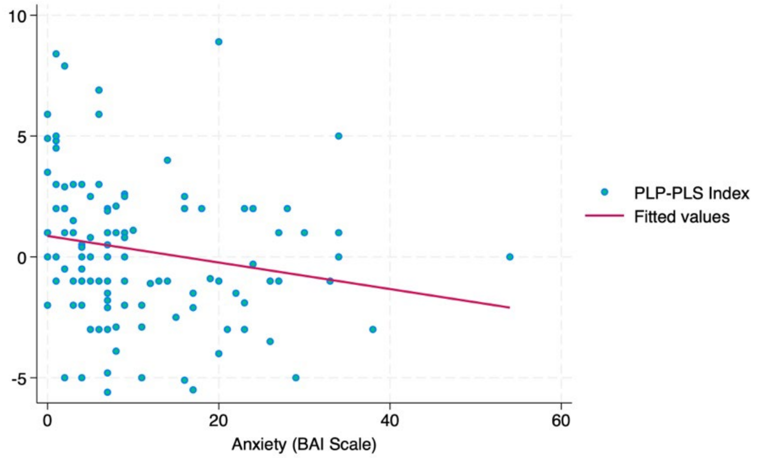

Supplementary Figure S9. Scatter plot of PLP-PLS index by anxiety (BAI Scale), adjusted using the final multivariable linear regression model.

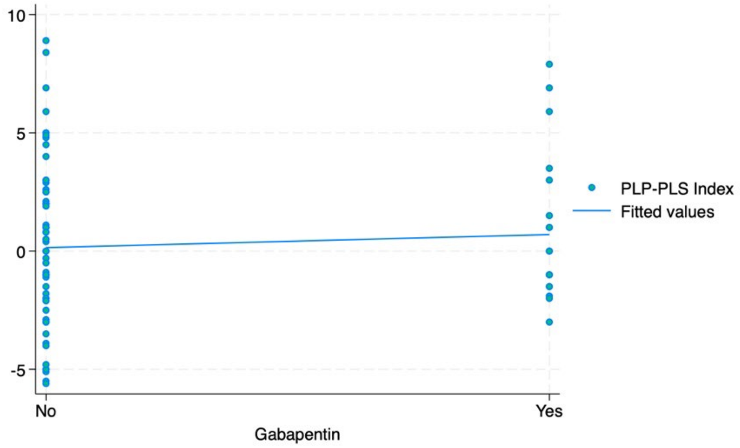

Supplementary Figure S10. Scatter plot of PLP-PLS index by gabapentin, adjusted using the final multivariable linear regression model.
